# Polyamide 12 Materials Study of Morpho-Structural Changes during Laser Sintering of 3D Printing

**DOI:** 10.3390/polym13050810

**Published:** 2021-03-06

**Authors:** Gražyna Simha Martynková, Aleš Slíva, Gabriela Kratošová, Karla Čech Barabaszová, Soňa Študentová, Jan Klusák, Silvie Brožová, Tomáš Dokoupil, Sylva Holešová

**Affiliations:** 1 Nanotechnology Centre, CEET, VŠB-Technical University of Ostrava, 17. listopadu 15/2172, 70800 Ostrava, Czech Republic; gabriela.kratosova@vsb.cz (G.K.); karla.cech.barabaszova@vsb.cz (K.Č.B.); sylva.holesova@vsb.cz (S.H.); 2 IT4 Innovations, VŠB-Technical University of Ostrava, 17. listopadu 15/2172, 70800 Ostrava, Czech Republic; 3 Institute of Transport, Faculty of Mechanical Engineering, VŠB-Technical University of Ostrava, 17. listopadu 15/2172, 70800 Ostrava, Czech Republic; ales.sliva@vsb.cz; 4 Department of Chemistry, VŠB-Technical University of Ostrava, 17. listopadu 15/2172, 70800 Ostrava, Czech Republic; sona.studentova@vsb.cz (S.Š.); jan.klusak.stk@vsb.cz (J.K.); 5 Department of Non-Ferrous Metals, Refining and Recycling, VŠB-Technical University of Ostrava, 17. listopadu 15/2172, 70800 Ostrava, Czech Republic; silvie.brozova@vsb.cz; 6 One3D s.r.o., Jižní 1443/29, 78985 Mohelnice, Czech Republic; tomas.dokoupil@one3d.cz

**Keywords:** PA12, structure, morphology, 3D print

## Abstract

The polyamide (PA)-12 material used for additive manufacturing was studied in aspects of morphology and their structural properties for basic stages received during 3D laser printing. Samples were real, big-scale production powders. The structure of polymer was evaluated from the crystallinity point of view using XRD, FTIR, and DSC methods and from the surface properties using specific surface evaluation and porosity. Scanning electron microscopy was used to observe morphology of the surface and evaluate the particle size and shape via image analysis. Results were confronted with laser diffraction particles size measurement along with an evaluation of the specific surface area. Fresh PA12 powder was found as inhomogeneous in particle size of material with defective particles, relatively high specific surface, high lamellar crystallite size, and low crystallinity. The scrap PA12 crystallinity was about 2% higher than values for fresh PA12 powder. Particles had a very low, below 1 m^2^/g, specific surface area; particles sintered as twin particles and often in polyhedral shapes.

## 1. Introduction

Additive manufacturing (AM), or rapid prototyping, solid freeform fabrication, or commonly used 3D printing, is one of the fastest evolving technologies, which has seen exponential growth mainly in the last decade. This technology’s basic principle is to compact materials to fabricate objects from 3D design data, layer by layer, firstly described by C. Hull 1986 [1]. These procedures can be applied to all types of additive manufacturing. Additive manufacturing offers several advantages comparing to other manufacturing technologies, such as creating objects with complex internal structures in a cost-effective way that does not require any special tools, molds, or assembly in a single manufacturing step [2]. It can reduce time and material consumption for a part’s production while maintaining mechanical behavior. A wide range of materials is possible to process by AM technology like paper, photopolymers, plastics, rubbers, metals, or ceramics [3]. There are many AM techniques, which differ in materials suitable to process, 3D printer construction, and in the principle of the technology itself. The International Organization for Standardization (ISO)/American Society for Testing and Materials (ASTM) 52900:2015 standard classify AM processes into seven categories: (1) material extrusion; (2) vat polymerization; (3) binder jetting; (4) material jetting; (5) direct energy deposition; (6) sheet lamination; and (7) powder bed fusion (PBF) processes [4]. A part of the PBF category and one of the most recognized AM method is selective laser sintering (SLS). The technology was developed by Carl Deckard and was based on a neodymium-doped yttrium aluminum garnet (Nd:YAG) laser with a power of 100 W [5]. Modern SLS 3D printers usually use a CO_2_ laser as a heat source to selectively melt powdered materials, aiming the laser automatically at the point in space defined by a 3D model, sintering the small powder particles together to create a solid object. The SLS printing process consists of several steps. Pre-heating the powder bed just below a polymer’s melting or softening temperature is the first step. Polymer particles are then spread in a thin layer by nozzles to the building platform. The third step is selective sintering or melting of powdered material by a laser according to computer-aided design (CAD) software trajectories. After this step, the building platform is placed lower, and nozzles spread the following polymer powder layer. The process is repeated until a full 3D print is finished [6]. One of the most significant advantages of the SLS method over other 3D printing techniques is the possible production of fully functional prototypes with the same mechanical properties as conventionally produced parts by injection molding or milling. Plastic powders, semi-crystalline or amorphous, are widely used materials in the SLS technique. Among the semi-crystalline polymers, polyamides are the most popular for SLS, where polyamide 12 dominates the production for the capability of generating substantial components for typical applications products [7]. Parts produced using polyamide 12 powders have superior mechanical and thermal properties. However, a polyamide powder’s volume ratio translated to the printed object is very small compared to waste. Usually, only 5–15% of total plastic powder consumption is used for finished part production. The remaining 85–95% of the powder deteriorates in physical and chemical properties due to thermal stress during pre-heating, sintering, and cooling [8]. Therefore, repeated usage of a previously processed powder material is necessary for economic and ecologic aspects SLS 3D printing and is usually performed by mixing with a virgin polymer powder. However, this re-using of previously processed materials gradually reduces the quality of the input material, and thus, the quality of the printed part is affected [9].

Improvements in the quality of finished parts and lessening in manufacturing time require an understanding of fine particles’ mechanical behavior in creating the layers of powders deposited during the manufacturing process. The polycondensation significantly influences the crystal structure, chain configuration, melting and crystallization behaviors, and rheological properties that are reflected in the macro-performance. A proper amount of end-capping agent is essential to decrease the signs of aging [10].

In this work, various stages of sintering of the polymer samples dedicated for the laser 3D printing process was studied to determine the differences in the structure, thermal behavior, morphology of the surface, and particle size and shape. Focus is given to real samples from production; therefore, even recycled and refreshed powders are given the same attention as for two edge samples—the very fresh powder, not used in any process, and the PA residue powder just after laser sintering. The topic is very important for R&D in manufacturing companies; hence, careful referencing to works available until now is performed. A discussion of the role of individual technological steps on the powder’s quality for printing is provided to give guidance to industrial applications.

## 2. Materials and Methods

Polyamide 12—PA 12 (as PA 2200) is one of the most resourceful materials in professional 3D printing. For its mechanical parameters, flexibility, and heat resistance, it is a great option for functional prototypes or end-use parts. Laser printing PA 12 requires no funding structures and thus enables printing the most intricate designs. The melting point in the range of 178–180 °C for nylon 12 (C_12_H_23_NO) is the lowest among the key polyamides. PA12 mechanical properties (hardness, tensile strength, and resistance to abrasion) are comparable to those of nylon 6/nylon 66. Low water absorption and density, 1.01 g/cm^3^, result from its fairly long hydrocarbon chain length, which causes its dimensional stability and a nearly paraffin-like structure. Nylon 12 is chemical resistant and not sensitive to stress cracking [11]. 

Various stages of nylon PA 2200 rising during 3D printing process were analyzed. Following labels are given to individual conditions of material: fresh PA12 (initial input material for 3D printing), scrap PA12, recycled PA12 (residual powder after 3D printing which was sieved) and renewed PA12 (mixture of fresh and recycled PA 12 in 1:1 ratio, also usable input material for 3D printing).

Several analytical methods were selected to get information about the morphology, particle size, and structure of all stages of the PA powders during the printing process.

The scanning electron microscope (SEM) JEOL JSM-7610F+ was utilized to examine the morphology of the polymeric surface. The various detectors were utilized to capture pictures of the surface but mainly back-scattered electrons (BSE) mode based on materials composition (so called COMPO mode). Due to the sample sensitivity to electrons and charging the samples were coated with a 20 nm layer of Pt.

The particles size image analysis (PSIA) based on SEM images was performed using J–Microvision software. Selected areas of samples’ SEM images were processed in a software environment, and the obtained data were postprocessed in ORIGIN 2019 software using for normality data testing to obtain mean values of particle size (PS).

The particle size evaluation and particle size distribution (PSD) was measured using a laser diffraction particle size (LDPS) analyzer (HORIBA LA-950 instrument) equipped with two 405 nm short-wavelength blue laser and one 650 nm red light laser source in combination with forward- and backscatter detection. The particle size analyses were directed with the refractive indices 1.60 (for PA12 samples) and 1.33 (for water medium). The PSD data (with the respect to symmetrical nature PSDs) were used for calculation of the specific surface area (SSA_cal_).

Specific surface area (SSA) was measured using Thermo Scientific Surfer employing liquid nitrogen adsorption. Prior to measurements, the samples were degassed at vacuum (10–6 bar) at 40 °C for 24 h. The SSA was calculated applying the BET (Brunauer–Emmett–Teller) equation assuming the area of the nitrogen molecule was 0.1620 m^2^. The experimental samples were dried before measurement at 40 °C for one day.

X-ray diffraction analysis, diffractometer Ultima IV Rigaku, reflection mode, working conditions CuKα radiation—40 kV, 40 mA; K—beta filter; CBO selection slit—BB; scintillation counter; continuous scan; scan speed/duration time—4°/min; step width—0.05°; scan axis—2Θ; scan range—5–60° 2Θ; incident and receiving slit 1—2/3°; receiving slit 2—0.6 mm.

The infrared spectra of PA 12 samples were measured using ATR (attenuated total reflectance) technique. The samples were put on the single-reflection diamond ATR crystal and pressed using a pressure tool. The infrared spectra were collected using FT-IR spectrometer Nicolet iS50 (ThermoScientific, Waltham, MA, USA) with DTGS detector on Smart Orbit ATR accessory. The conditions of measurement were: spectral region 4000–400 cm^−1^, spectral resolution 4 cm^−1^; 64 scans; Happ–Genzel apodization.

The melting and crystallization behaviors of the PA 12 samples were determined using Setaram DSC131 Evo DSC thermal analyzer. Samples about 7 mg were analyzed under argon atmosphere. After equilibration at −20 °C for 10 min, the samples were heated at a rate of 10 °C/min to 300 °C, kept at 300 °C for 10 min, and then cooled down to 25 °C again at a rate of 10 °C/min.

## 3. Results and Discussion

### 3.1. Morphology and Particle Size Observation

One of the crucial parts of the 3D printing technology for polymers is the size and shape of powders used for production [12,13]. A strong objective technique for relevant information about the state of a powder is microscopy image examination of the powder particles. Not only can the objects’ diameters in local areas be obtained but also the real character of the particles’ morphology. In this chapter, we focus on the observation of the particles’ surface, defects, or their anomalies, carefully comparing with the state of art.

By observing the character of the studied particles, two phenomena were identified. Particles had a relatively smooth surface, and numerous particles with cracks were detected. The shape of the powder particles was mostly ellipsoidal with deformations in one of the dimensions. The observation of cracks on fresh PA12 particles is in contrast to the work [8,14] where authors state “The new powders have a good spherical morphology, whereas the aged or extremely aged powders have irregular shapes with cracks or gaps on the surface” but in good accord with study [14].

**Figure 1:**
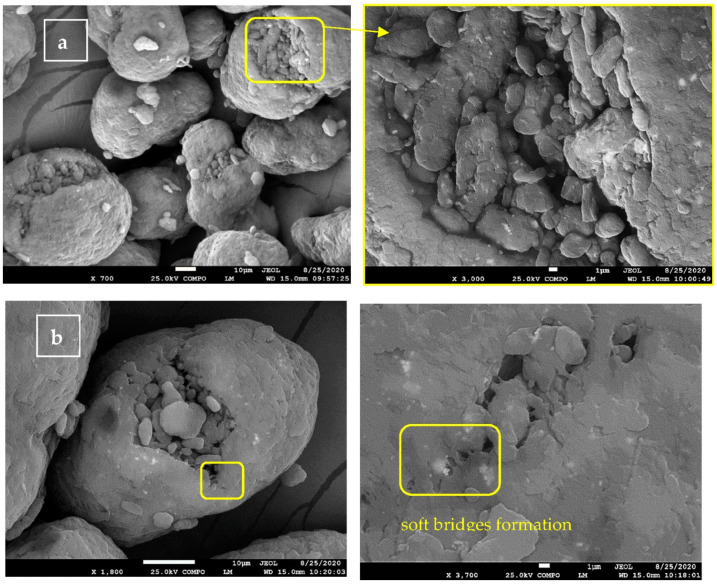

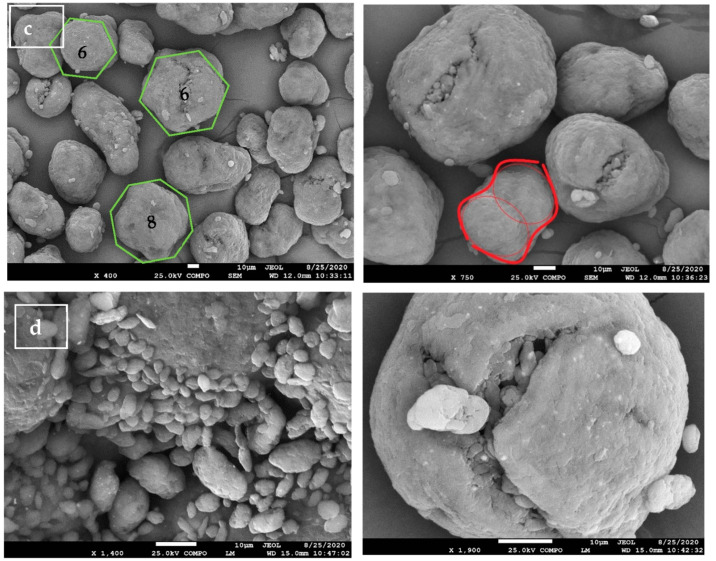
Scanning electron microscopy (SEM) images of experimental samples (**a**) fresh polyamide (PA)-12 and detail of crack; (**b**) scrap PA12 and crack detail with melted bridges on the edge of crack in detail; (**c**) recycled PA12 particle with observed polyhedrons and (**d**) renewed with detail sample. Images captured at 25 kV in COMPO mode. This is a figure. Schemes follow the same formatting.

The surface of fresh PA12 sample particles (Figure 1a) was composed of flakes with the noticeable edges and layered character. The cracks were visible; however, a hollow character of particle was not observed as the external shell part and fine inner granular matter. Those fine particles appeared in the bulk of the polymeric powder. 

Particles after laser processing all three remaining samples, had smoother a melted-like surface character that can be connected to the conditions of the printing process [15]. The temperature of printing condition changes the character of cracks, and visible thin bridges are observed on the cracks’ ends (Figure 1b detail in yellow rectangle). The particles of sample recycled PA12 (Figure 1c) consist of the same particles as scrap PA12 (Figure 1b) particles; however, the uniformity regarding the size achieved via the sieving process is evident. The sample of renewed PA12 (Figure 1d) contained places where a relatively high concentration of small particles less than 10 μm size flocked to the common big particles was observed. This phenomenon might be caused by electrostatic forces created during renewing the PA12 powder, where mixing of recycled and fresh powder is taking place. “For a polymer particle of diameter about 10 µm (i.e., *R* = 5 µm) resting on a rigid surface, the magnitude of van der Waals forces may vary from 10 to 500 nN depending upon the particle surface properties” [16]. The electrostatic interactions with significantly charged dielectric particles may considerably affect the apparent cohesion of the powder material, where the electrostatic image force can also be a key participant.

After laser sintering, several shapes were redefined, and some particles were observed as polyhedrons. The shape effect was demonstrated on a recycled PA12 sample, as the most refined powder, in the sense of uniformity of particles, was obtained via a sieving process (Figure 1c). Except for reshaping of the preliminarily relatively random shape of the particles, we observed the two polymer particles sintering phenomenon that was modelled by Belemans et al. [17]. 

The powder particles were fused jointly under the impact of a laser beam (Figure 1c right). To obtain good quality material properties in the definitive product, the powder particles need to create a homogeneous melt throughout the production process. The simulations uncovered that an ideal sintering process requires a low ambient temperature, a narrow laser beam width with sufficient power to heat the powder particles a few degrees over the melting temperature, and a polymer of which the viscosity decreases suggestively within these few degrees [17].

**Figure 2:**
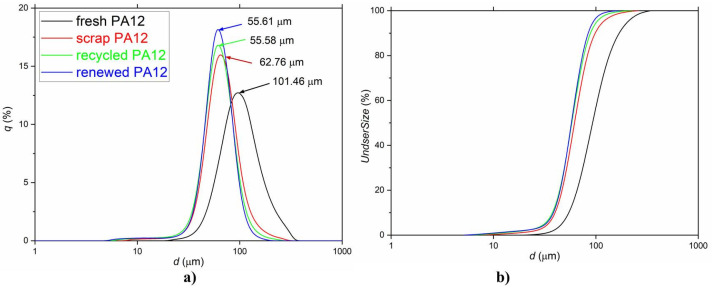
Log-normal (**a**) and cumulative (**b**) particles size distribution (PSD) of experimental samples. The mode diameter (dm) values are noted in the graph.

All findings from the above discussed image microscopy observation were confronted at particles size examination using laser diffraction methods. The particle size distributions (PSD) curves (obtained using LDPS analysis) of experimental samples with marked mode size (dm, the most common particle diameter in the sample volume) are shown in Figure 2. Particle size parameters as volume weighted mean diameters (d_43_), diameters d_10_ and d_90_ (representing 90% of the distribution is below d_90_, and 10% of the population is below d_10_) are summarized in Table 1. The PSD data (with the respect to symmetrical nature PSDs) were used for numerical evaluation of the specific surface area (SPA). SPA values (Table 1) are indicative for the comparison with measured SSA_BET_ values.

All experimental samples showed monomodal particle size distribution (Figure 2a). The renewed PA 12 sample had the narrowest PSD in the interval value 39–84 µm with a mode diameter of 55.61 µm and in opposite the widest PSD curve had fresh PA 12 sample with mode diameter 101.46 µm and interval 54–168 µm. With the respect to the most common average value of particles in the sample, the volume can be arranged by weighted mean diameters (d_43_) as follows: scrap PA 12 > recycled PA 12 > renewed PA 12 > fresh PA 12.

Very interesting characteristic is specific surface area (SSA), where the effect of sintering–melting and transformation is causing radical reduction of surface area up to 10 times (Table 1).

Specific surface area (SSA_cal_) values are calculated from the particle size distribution measurement using the laser diffraction technique [18] and using material parameters such as material density ρ, relative volume for the particle size class *V_i_*, particle size *d_i_*, and mean diameter *d*_43_. For the SPA values are mentioned in Equation (1) [18]:

(1) $$SSAcal=\frac{6\sum^{\;}\frac{V_{i}}{d_{i}}}{\rho \sum^{\;}V_{i}}=\frac{6}{\rho d_{43}}$$

From the measured SSA_BET_ values (Table 1), it is evident that all samples showed a small surface area (<10 m^2^/g). The surface of the scrap PA 12 and recycled PA 12 samples showed insufficient sensitivity to nitrogen surface adsorption which was reflected in the SSA_BET_ values smaller than 1 m^2^/g. For this reason, the SSA_cal_ values have a better predictive value. From these values, it can also be seen that a fresh PA 12 sample will show the highest specific surface area, followed by renewed PA 12 and recycled PA 12 samples, and the lowest specific surface area can be assumed for scrap PA 12 samples.

**Figure 3:**
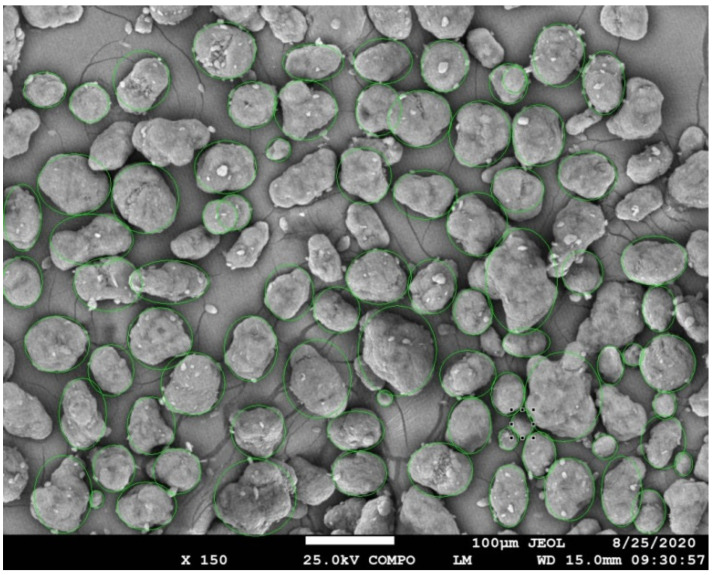
Example SEM image of analyzed material. Particle size image analysis, where all shapes are approximated as round circle-equivalent or oval.

To correlate the results of the LDPSA, SEM image analysis (PSIA) was performed. A large number of particles’ diameters was processed to obtain the statistical distribution of particle size from the SEM images (Figure 3). Since the shapes of the particles were not regular, the width (shorter diameter) of particle and the length (longer diameter) of particle were taken into account for the analysis (Table 2).

**Figure 4:**
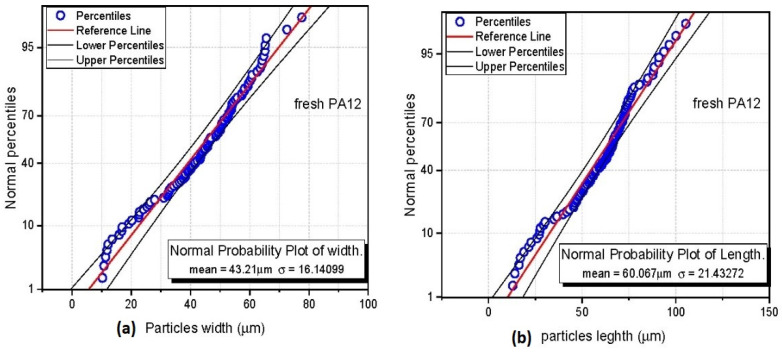
Example of normal probability plots for fresh PA12 sample (**a**) width; (**b**) length measurement.

The data analysis was conducted in the statistical environment of the software Origin (Figure 4) as follows: input data was arranged from smallest to largest, and then the serial number of the sorted data was generated using the following method. Blom plotting: position method (*i*, *n*) = (*i* − 0.375)/(*n* + 0.25), where *i* is the serial number and n is the total number of the non-missing input data.

(2) $$p\left(x\right)=\frac{1}{\sigma \sqrt{2\pi }}\exp(-{\frac{\left(x-\mu \right)}{2\sigma^{2}}}^{2})$$

*p*(*x*) is density function for normal distribution; μ is mean of normal distribution, the location parameter; σ is standard deviation of normal distribution, the scale parameter.

To get the relevant comparison of particle size from both methods (LDPSA and PSIA), we consider particle length for the SEM image analysis method because laser diffraction is designed to measure the largest diameter of the analyzed particle. The distribution curve of fresh PA 12 represents a wide peak for LDPSA, and sigma deviation in PSIA is highest, indicating a broad range of sizes of the fresh powder particles. The sample of recycled PA12 had the smallest average particle size because of compression during sintering and subsequent sieving of the powder. 

### 3.2. Phase Analysis and Structure Parameters

All technological stages of powdered PA12 were investigated using XRD methods. Phase analysis using the database JCPDS ICCDD PDF 2011 was performed, where PA12 referred to Nylon 2/12/2, 10 (C_26_H_48_N_4_O_4_)n 147: P-3, card 00-057-1433.

**Figure 5:**
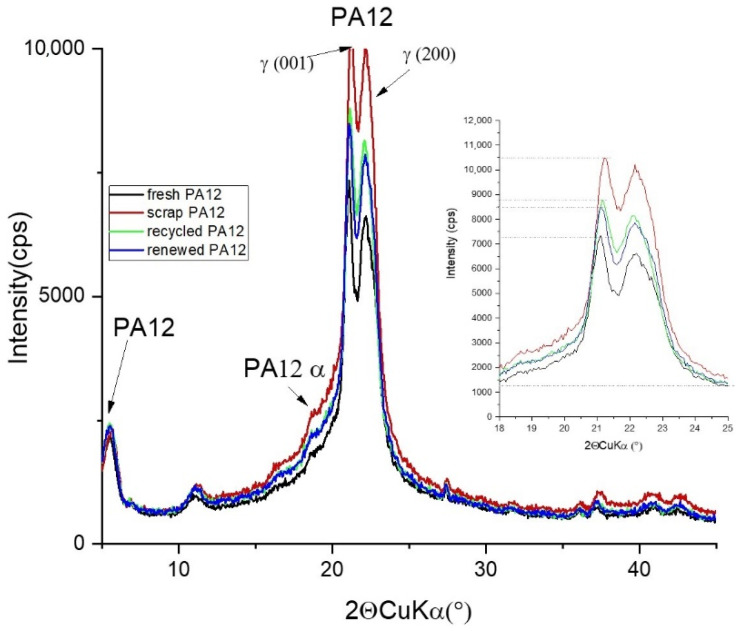
X-ray diffraction (XRD) patterns of all studied samples in range 3–45° 2Θ with evaluated α and γ phases of polyamide 12 and detail of studied main γ-PA diffractions in range 18–25° 2Θ.

X-ray diffraction patterns are shown in Figure 5, showing the polymorphism and crystalline structure alterations of weakened PA12 powders. PA12 can be crystalized in structures of α and γ phases, where the major γ phase acts as a stable structure. PA12 chains in the α phase are oriented in an anti-parallel way with stretched trans chain conformation, while in the γ phase chains are oriented in a parallel way with twisted helical conformation around amide groups [19]. The unit cell of the γ phase of polyamide 12 is monoclinic with dimensions a = 0.935 nm, b = 3.22 nm (fiber identity period), c = 0.487 nm. The unit cell has four [–NH(CH_2_)_11_CO–] repeating units. The density of the polyamide 12 crystal calculated from the unit cell is 1.04 g/cm^3^ [20]. As for the fresh PA-12 powder, two peaks, d(001) = 0.421 nm and d(001) = 0.401 nm, characterize the γ phase of PA12; weak reflections at d = 0.479 and 0.392 nm are the α phase. 

Intensity of the peaks is changing during laser sintering process. The relative intensity of fresh PA12 is the lowest of all samples, in contrast to the scrap PA12 sample, where the intensity of both peaks of the γ phase is highest (Table 3). To the intensity progression the findings from the calculation of crystalline thickness size correspond well. For the calculation of mean crystallite size, the Williams–Hall approach using PA 12 (001) and (200) basal diffractions was utilized.

The interlayer space of both observed highest diffractions did not change much with respect to position; however, the width of peak (001) is narrowest for fresh PA12 as well as renewed PA12 compared to scrap PA12 sample.

The least ordered material in the sense of lamellar crystallite size is the scrap PA12 material giving Lc equals 4.2 nm, while fresh PA12 is highly ordered at 6.0 nm with the lowest intensity of the peak. This effect can be explained via average crystalline lamellar thickness Lc and the amorphous layer thickness La (not calculated here).

**Figure 6:**
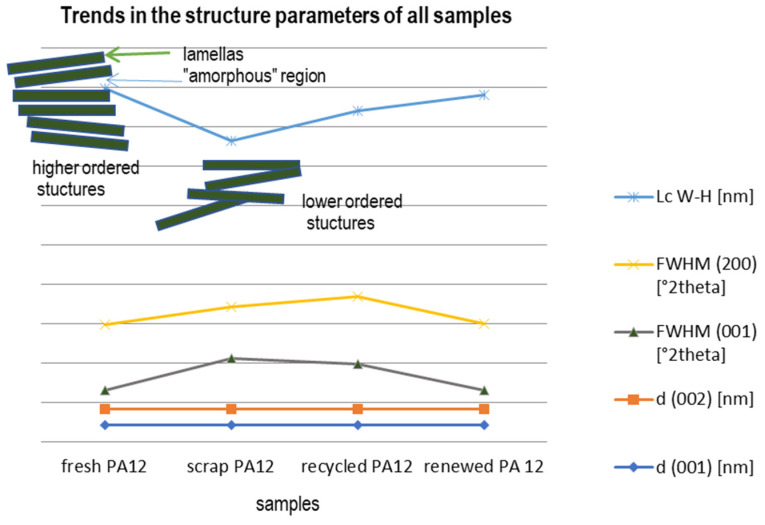
Charts explaining the tendency of selected calculated and measured parameters of material structure of samples with demonstration of lamellar structure ordering of PA12 regarding mean crystallite size Lc.

The development of the crystalline thickness for the period of cooling and heating indicates the occurrences of partial surface crystallization and softening. The transition from the γ to the α phase with rising temperature occurs from a “one-dimensional-melting” type of separation between the hydrogen-bonded sheets [21]. The partial phase transformation from γ to α may be the reason for the decrease in Lc due to disruptions caused in the γ lamella stacking continuum (Figure 6).

### 3.3. FT Infrared Spectroscopy Analysis

**Figure 7:**
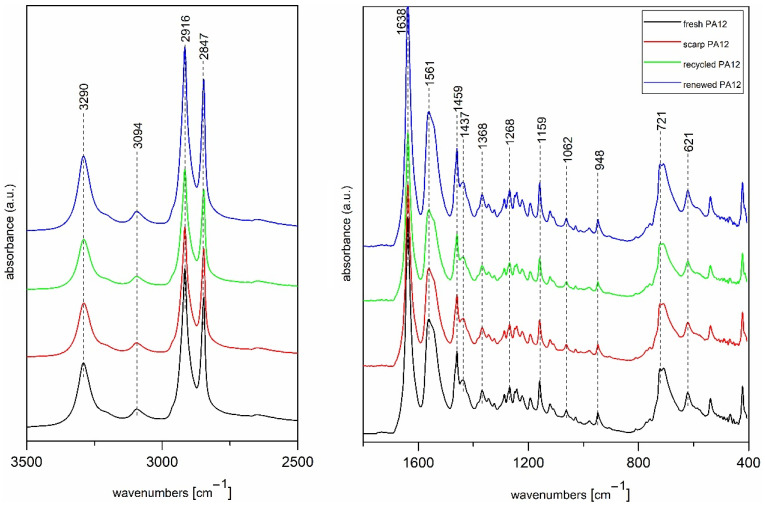
Fourier transform infrared (FTIR) spectra of PA 12 samples: fresh PA 12, scrap PA 12, recycled PA 12, and renewed PA 12.

The Fourier transform infrared (FTIR) spectra of PA12 samples are shown in Figure 7 together with characteristic infrared bands and their roles which are summarized in Table 4.

The absorption band at 3290 cm^−1^ is assigned to N-H stretching vibration together with shoulder at 3094 cm^−1^ which belongs to Fermi resonance of the *ν* (N-H) stretching. Two intensive peaks at 2916 and 2847 cm^−1^ correspond to asymmetric and symmetric CH_2_ stretching vibrations. The characteristics bands for amides are at 1638 and 1561 cm^−1^ which are so-called Amide-I and Amide-II modes, together with band at 1268 cm^−1^ corresponded to Amide-III and 621 cm^−1^ to Amide-IV [19,22]. Amide bands as hydrogen-bonded N-H stretching vibrations, Fermi resonance of *ν* (N-H) stretching band, Amide-I and Amide-II modes are sensitive to aging and can be treated as character reference to determine the chemical alterations during laser sintering. Interestingly, as we can observe from these spectra (Figure 7) the intensities and positions of these bands are almost the same, so we can conclude that sintering process had no impact on chemical composition of PA12 powders. Moreover, the most common chemical degradation of polymers is oxidation and in polyamides, CH bonds of the methylene groups adjacent to the nitrogen are the weakest bonds, therefore this carbon undergoes oxidation. Therefore, we can also examine changes of peaks at 1368 cm^−1^ corresponded to δ (CH_2_) twisting vibration, 1159 and 1062 cm^−1^ to skeletal motion involving CO-NH groups, and 946 cm^−1^ to in-plane deformation of CO-NH [21]. However, in this case there aren’t visible changes of these peaks in the infrared spectra for all measured samples (Figure 7) compared to examples of aged samples mentioned in literature [7,19].

### 3.4. Thermal Characterization

**Figure 8:**
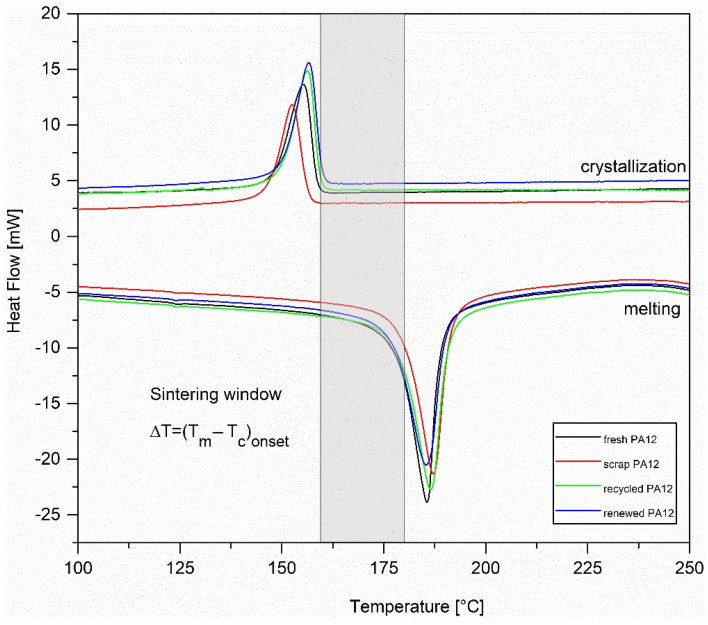
DSC thermograms of PA12 samples: fresh PA12, scrap PA12, recycled PA12, and renewed PA12.

Thermal characteristics for PA12 powders (heating and cooling cycles) tested by DSC are shown in Figure 8 and Table 5. For all powders, the heating curves display sharp single endotherms with melting points at 185−187 °C which correspond to γ crystal forms. A small amount of α phase confirmed by the XRD technique was not identified by DSC because of rapid α-to-γ transfer which is occurring on heat treatment near the melting point [23]. The sintering window as well as onset melting temperatures is the important information for the parameter setting of SLS printing, especially the preheating temperature which should be close to the onset melting temperature. As we can observe from Table 5, renewed PA12 has a narrower sintering window than fresh PA12; this is unfavorable for the process of SLS printing. In the case of recycled PA12, the sintering window is almost the same as the one for fresh PA12. After the heating cycle, the rise of the melting temperatures, melting enthalpies, and crystallinity of scrap PA12 and recycled PA12 samples, compared to fresh PA12, originates from the post-crystallization [24]. The values T_m_, ∆H_m_, and *Χc* for renewed PA12 then correspond to the fact that it is a mixture of fresh and recycled PA12.

## 4. Conclusions

Reviewing all analyses performed during the study, the following important facts evolved:

Effect of size/ shape and morphology: Fresh PA 12 powder exhibited a wide range of particles size, where small particles below 10 μm as well as broken large particles were assessed. Small particles bore high (up to 500 nN) electrostatic forces helping optimal laser sintering. The surface of the particles was in some way rough, and shapes were randomly ellipsoidal, which is a great predisposition to obtain optimal sintering conditions; the highest SSA of the studied samples was 6.7 m^2^/g, which represents a porous and lumpy surface. During sintering, the powder gets more compact, and the specific surface area decreases to less than 1 m^2^/g. Apparently, the porosity decreased as well, while the distribution curve of the particle size became narrow, and the average size of particles was generally big. Shapes got smore pronounced as polyhedral complex object, and details of particle defects were observed, such as many clear melt bridges on the edges of cracks.

Structural changes: Evaluating the structure was limited to the changes in the ordering of materials because variation in chemistry and bonds was not observed using FTIR. The powders examined before and after laser sintering revealed a decrease in lamellar crystallite size of the sample at XRD method. The partial phase transformation from γ to α may be the reason of decreasing Lc due to disruptions caused in the γ lamella stacking continuum. Using DSC, the scrap PA12 crystallinity was higher by about 2% than values for fresh PA12 powder. Here, we have to differentiate the nuances in crystallinity evaluation. XRD considers the lamellar structure measuring the crystalline thickness of the as-received sample perpendicularly to lamella, while DSC works with the changes initiated during the heat treatment. Therefore, we can presume enhanced crystallinity during laser sintering, where the amorphous phase is less pronounced. From a technical point of view, it is required to keep the process temperatures between the melting and crystallization points of the material. Material degradation is reliant on the laser energy applied, where high values enhance deterioration. On the contrary, low values prevent particle blend and reduce the mechanical strength of printed parts. Therefore, scrap PA12 material becomes less effective after several recycling cycles.

## Figures and Tables

**Table 1:** Particle size (d_43_, d_10_, and d_90_) and specific surface area (SSA_BET_, SSAcal) of experimental samples.

Samples	d_43_	d_10_	d_90_	SSA_cal_	SSA_BET_
	(µm)	(µm)	(µm)	(m^2^/cm^3^)	(m^2^/g)
fresh PA 12	92.8	54.5	168.4	0.7010	6.7
scrap PA 12	66.9	40.2	97.9	0.1057	0.7
recycled PA 12	61.3	37.9	87.6	0.1158	0.4
renewedPA 12	59.6	38.9	83.8	0.1183	2.2

**Table 2:** Particle size values obtained from image analysis obtained from statistical analysis regarding the width and length of particles.

Samples	Width Mean (μm)	Standard Deviation	Length Mean (μm)	Standard Deviation
fresh PA12	43.2	16.14	60.07	21.43
scrap PA12	38.91	14.05	48.93	18.71
recycled PA 12	37.72	13.55	47.35	17.10
renewed PA 12	40.35	13.38	53.06	17.47

**Table 3:** Structural parameters of studied samples, interlayer space d of two basal diffractions and their full width at half maximum FWHM as well as mean crystallite size were calculated according to the Williams and Hall approach Lc _W-H._

	d (001) (nm)	d (200) (nm)	FWHM (001) (°2Θ)	FWHM (200) (°2Θ)	Intensity (001)	Lc _W-H_ (nm)
fresh PA12	0.4219	0.4004	0.48	1.66	6048	6.0
scrap PA12	0.4206	0.4001	1.30	1.303	9275	4.2
recycled PA12	0.4223	0.4006	1.15	1.72	7521	4.7
renewed PA 12	0.4223	0.4007	0.47	1.70	7238	5.8

**Table 4:** Characteristic infrared bands and their assignments of PA12 samples.

Vibrational Frequency [cm^−1^]	Assignments
3290	*ν*(N–H) stretching
3094	Fermi resonance of *ν*(N–H) stretching
2916	*ν*(CH_2_) asymmetric stretching
2847	*ν*(CH_2_) symmetric stretching
1638	Amide-I (*ν*(C=O) stretching and *ν*(C–N) stretching)
1561	Amide-II (δ(N–H) bending and *ν*(C–N) stretching)
1459	*δ*(CH_2_) scissoring
1368	*δ*(CH_2_) twisting
1268	Amide-III (*ν*(C–N) stretching and δ(C=O) in-plane bending)
1159	Skeletal motion CO–NH
1062	Skeletal motion CO–NH
948	*δ*(CO-NH) in-plane bending
721	*ρ*(CH_2_) rocking
621	Amide-IV (δ(N–H) out-of-plane bending)

**Table 5:** Quantitative data of thermal properties for PA12 samples.

	Fresh PA12	Scrap PA12	Recycled PA12	Renewed PA12
melting point T_m_ [°C]	185.6	187.2	186.5	185.4
melting onset T_m_*^onset^* [°C]	178.0	179.7	178.7	176.9
melting enthalpy ∆H_m_ [J/g]	110.7	114.3	120.2	117.9
crystallization point T_c_ [°C]	155.3	152.6	156.3	156.6
crystallization onset T_c_*^onset^* [°C]	158.6	156.4	159.3	159.8
crystallization enthalpy ∆H_c_ [J/g]	−57.9	−50.6	−62.0	−59.6
Crystallinity *Χc* [%]	52.9	54.6	57.4	56.3
sintering window [°C]	19.4	23.3	19.4	17.1

Note: “sintering window” is the temperature interval between melting and crystallization onset points.

## Data Availability

The data presented in this study are available on request from the corresponding author.
